# Data in support of a functional analysis of splicing mutations in the *IDS* gene and the use of antisense oligonucleotides to exploit an alternative therapy for MPS II

**DOI:** 10.1016/j.dib.2015.10.011

**Published:** 2015-10-28

**Authors:** Liliana Matos, Vânia Gonçalves, Eugénia Pinto, Francisco Laranjeira, Maria João Prata, Peter Jordan, Lourdes R. Desviat, Belén Pérez, Sandra Alves

**Affiliations:** a Research and Development Unit, Department of Human Genetics, INSA, Porto, Portugal; b Department of Biology, Faculty of Sciences, University of Porto, Porto, Portugal; c Research and Development Unit, Department of Human Genetics, INSA, Lisbon, Portugal; d Biochemical Genetics Unit, Center for Medical Genetics Jacinto Magalhães, Porto Hospital Center, Porto, Portugal; e i3S - Instituto de Investigação em Saúde/IPATIMUP - Institute of Molecular Pathology and Immunology of the University of Porto, Porto, Portugal; f Centro de Diagnóstico de Enfermedades Moleculares, Centro de Biología Molecular Severo Ochoa, UAM-CSIC, Universidad Autónoma de Madrid, Madrid, Spain; g CIBER de Enfermedades Raras (CIBERER), Madrid, Spain; h IDIPaz, Madrid, Spain

## Abstract

This data article contains insights into the methodology used for the analysis of three exonic mutations altering the splicing of the *IDS* gene: c.241C>T, c.257C>T and c.1122C>T.

We have performed splicing assays for the wild-type and mutant minigenes corresponding to these substitutions. In addition, bioinformatic predictions of splicing regulatory sequence elements as well as RNA interference and overexpression experiments were conducted.

The interpretation of these data and further extensive experiments into the analysis of these three mutations and also into the methodology applied to correct one of them can be found in “Functional analysis of splicing mutations in the *IDS* gene and the use of antisense oligonucleotides to exploit an alternative therapy for MPS II” Matos et al. (2015) [1].

**Specifications table**

**Table:** 

Subject area	*Biology*
More specific subject area	*Molecular genetics*
Type of data	*Text, figures, tables and graphs*
How data was acquired	*Agarose gel images,* quantitative real-time PCR*, western blotting, in silico analyses (Splicing rainbow and ESEfinder 3.0 software)*
Data format	*Raw, analyzed*
Experimental factors	*Cell lines (Hep3B and COS-7) treated with different minigenes*
Experimental features	*Wild-type and mutant minigenes were transfected in COS-7 and Hep3B cell lines and the splicing patterns analysed by RT-PCR. SRSF1 (formerly ASF/SF2) siRNAs and plasmids coding for SRSF2 (formerly SC35), hnRNP E1 and hnRNP E2 splicing factors were transfected in Hep3B cells and the experiments analysed by quantitative real-time PCR and/or Western blotting. In silico predictions were done using Splicing rainbow and ESEfinder 3.0 software.*
Data source location	*INSA, Porto, Portugal*
Data accessibility	*All data provided within the article*

**Value of the data**

• Data show the methodology for the analysis of the effect of three exonic mutations on splicing.
• Experimental and *in silico* data are presented.
• The data may be valuable for future studies addressing the impact of mutations in splicing.

## 1 Data, materials and methods

The *IDS gene* encodes the lysosomal hydrolase iduronate-2-sulphatase, the enzyme that is deficient in the X-Linked Lysosomal Storage Disease; Mucopolysaccharidosis type II [2].

**Fig. 1:**
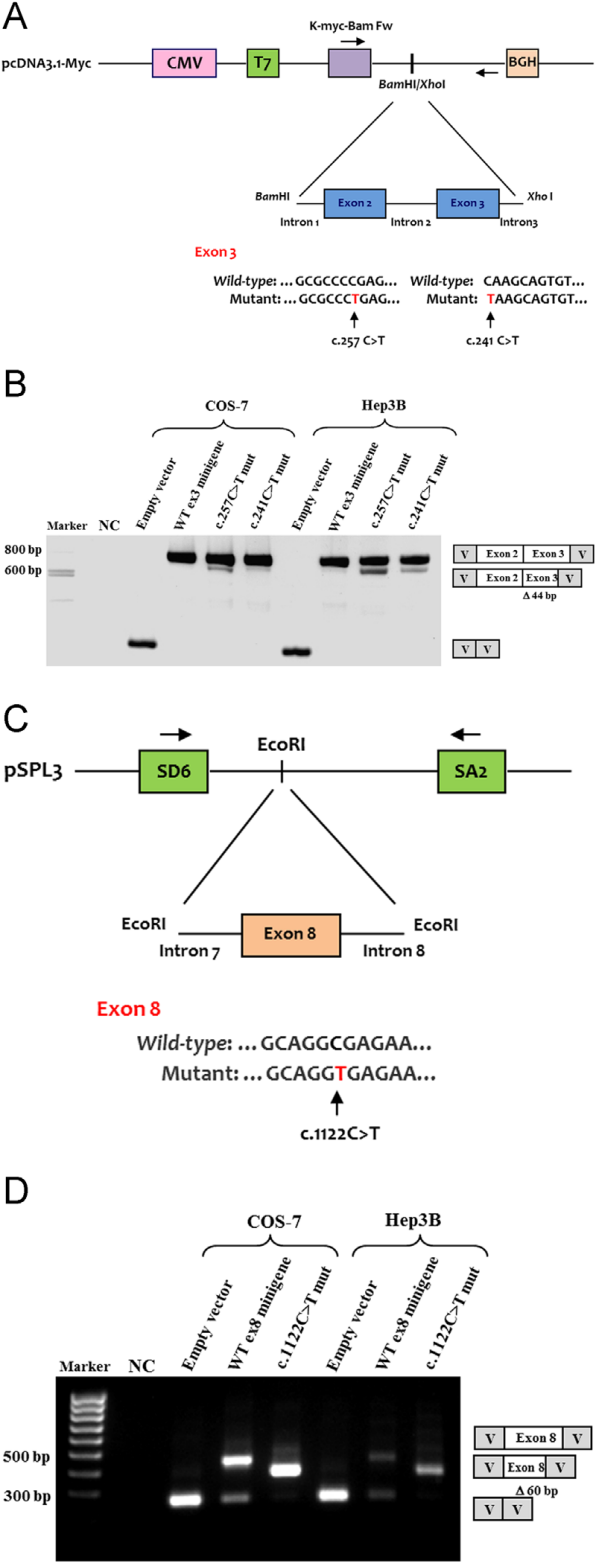
Splicing assays for the wild-type (WT) and mutant minigenes corresponding to the *IDS* nucleotide changes c.257C>T and c.241C>T in exon 3, and c.1122C>T in exon 8. (A, C) Diagrams of the reporter minigenes used in the functional splicing experiments. Normal and mutated genomic *IDS* sequences were cloned into the pcDNA3.1-myc or pSPL3 vectors to generate the indicated minigenes. Exons are shown by boxes and introns by straight lines. For all exonic alterations, the WT and mutant regions are shown and the specific changes marked by an arrow. (B, D) Wild-type and mutant minigenes were transfected into COS-7 and Hep3B cells and the splicing pattern analyzed by RT-PCR using the indicated vector-specific primers (arrows in diagrams A and C). Minigene expression of the splicing mutations in exon 3 (c.257C>T and c.241C>T) revealed two transcripts, a predominant one with exon 2 and a mutated exon 3, and a minor transcript of smaller size in which the first 44 nucleotides of exon 3 were missing. The WT minigene produced a single transcript of normal size (B). For the synonymous c.1122C>T change in exon 8, the mutant minigene showed a single transcript lacking the last 60bp of exon 8. The WT construct produced two bands, one corresponding to the transcript with exon 8 inserted, the other to a transcript resulting from an expected splicing event between the vector splice sites (D). A diagram of the bands characterized by sequence analysis is also provided. NC – negative control; V – vector sequence.

**Fig. 2:**
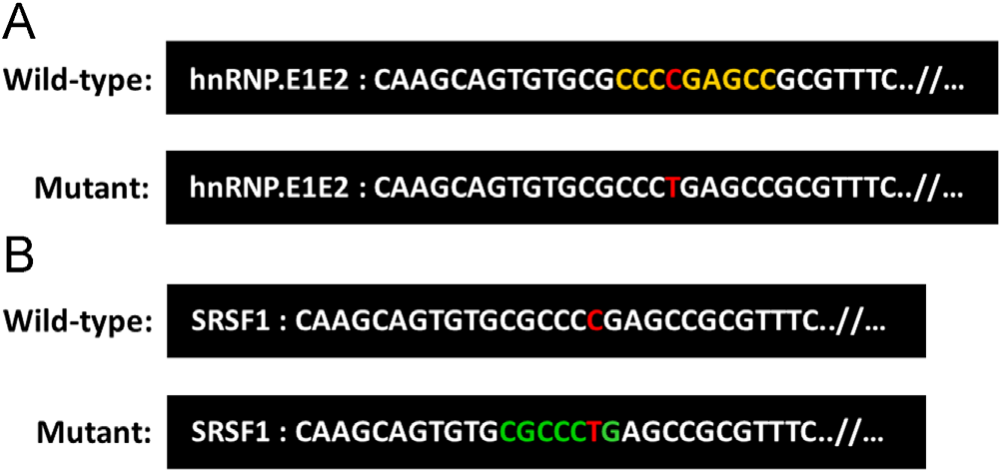
Western blot analysis of the degree of depletion achieved after transfection of siRNAs for luciferase (control) and SRSF1 (formerly ASF/SF2) plus the WT exon 3 or c.257C>T mutation minigenes. The immunoblot shows the suppression of the endogenous SRSF1 when silencing was performed using 200 µM of the siRNA pool. α-tubulin was used as a loading control.

**Fig. 3:**
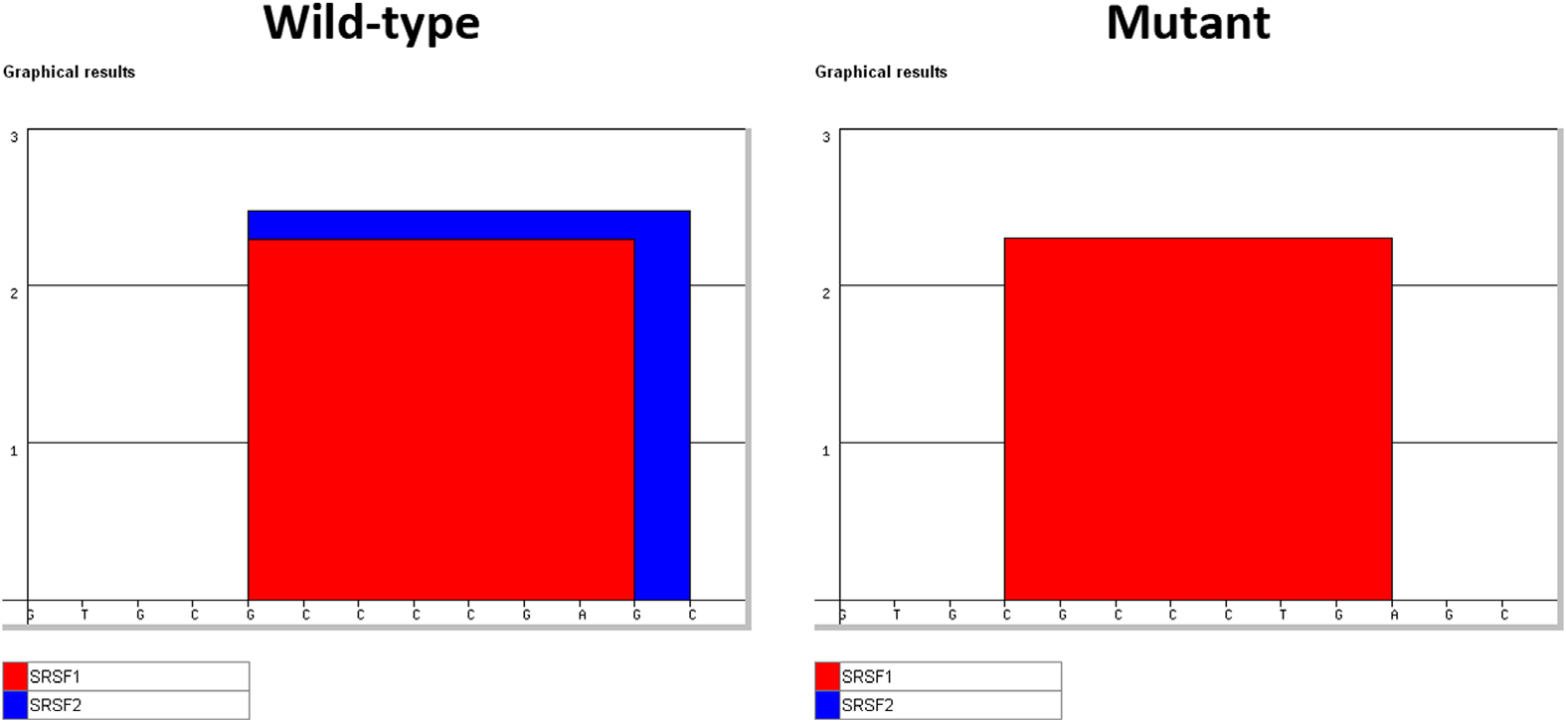
**:** Bioinformatic predictions of splicing regulatory sequence elements in *IDS* exon 3 using the *ESEfinder* 3.0 software. For the mutation c.241C>T, no alterations were predicted. In the presence of the c.257C>T mutation, a putative binding motif for SRSF1 (formerly ASF/SF2) is slightly altered (red square) and a binding motif for SRSF2 (formerly SC35) is eliminated (blue square).

**Fig. 4:**
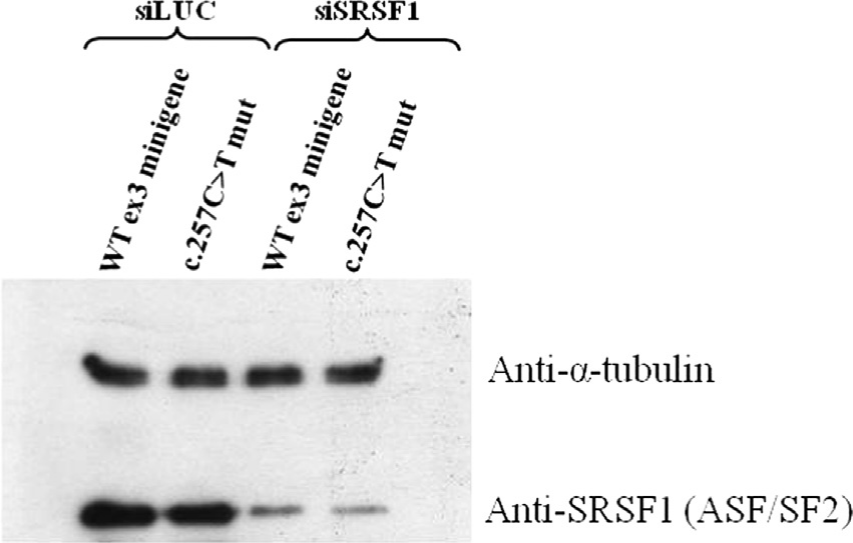
Western blot analysis of the degree of depletion achieved after transfection of siRNAs for luciferase (control) and SRSF1 (formerly ASF/SF2) plus the WT exon 3 or c.257C>T mutation minigenes. The immunoblot shows the suppression of the endogenous SRSF1 when silencing was performed using 200 µM of the siRNA pool. α-tubulin was used as a loading control.

Here, we performed cell-based functional splicing assays to deeper analyze the effects of two splicing mutations located in exon 3 of *IDS*, c.241C>T and c.257C>T and one in exon 8, c.1122C>T that were also studied in Matos et al. [1]. The pathogenic effects of these mutations are shown in Fig. 1. Also, all the data relative to oligonucleotides sequences used in the work are depicted in Table 1. Furthermore, to identify the putative SR proteins involved in the splicing regulation we have undertaken bioinformatic predictions of splicing regulatory elements (SREs) in the *IDS* exon 3 (where the mutations c.241C>T and c.257C>T are located) using *ESEfinder* 3.0 and Splicing Rainbow software (Fig. 2 and 3). Finally, we have conducted RNAi and overexpression experiments that were quantified by Real time PCR and Western blot (Table 1, Table 2 and Fig. 4).

### 1.1 Oligonucleotides sequences

See Table 1.

### 1.2 Minigenes construction and *in vitro* functional splicing analysis

For the *in vitro* splicing analysis of the variants c.257C>T and c.241C>T in *IDS* exon 3, the respective regions of patient and healthy control genomic DNA were amplified and cloned into pcDNA3.1-myc, a modified plasmid vector (Invitrogen, Carlsbad, USA) (Table 1 and Fig. 1A).

Also, to functionally investigate the splicing defects caused by c.1122C>T in exon 8, wild-type (WT) and mutant minigenes were constructed in vector pSPL3 (Exon Trapping System, Life Technologies, Gibco, NY, USA) (Table 1 and Fig. 1C). To perform the functional splicing assays, Hep3B and COS-7 cell lines (4×10^5^) were grown in 6-well plates and transfected with WT or mutant minigenes (2 μg) using 4 μl of Lipofectamine 2000 (Invitrogen, Carlsbad, USA). At 24 h post-transfection, total RNA was extracted from the cells and used as a template for cDNA synthesis. RT-PCR was then performed using vector specific primers (Table 1) and the amplified products were separated by agarose gel electrophoresis (Fig. 1B and D).

### 1.3 Bioinformatic analysis of SREs in *IDS* exon 3

*In silico* predictions for alterations in exonic splicing enhancer or silencer sequences (splicing regulatory elements – SREs) in the presence or absence of c.257C>T and c.241C>T mutations in *IDS* exon 3 were performed using *ESEfinder* 3.0 (http://rulai.cshl.edu/cgi-bin/tools/ESE3/esefinder.cgi?process=home) [3], [4], and *Splicing Rainbow* (http://www.ebi.ac.uk/asd) [5] software (Figs. 2 and 3).

**Figure:**
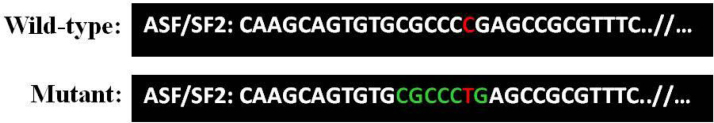

**Figure:**
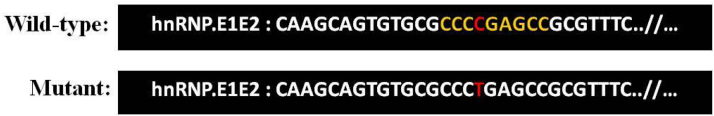

### 1.4 Quantitative Real time PCR and Western blot assays to confirm overexpression and depletion of splicing factors

To confirm the predicted changes in the splicing factors SRSF2 (formerly SC35), hnRNP E1 and hnRNP E2, overexpression studies were performed using plasmids coding for them which were co-transfected in Hep3B cells with WT or mutant c.257C>T minigenes. All transfections were performed using Lipofectamine 2000 reagent. At 48 h post-cotransfection, the cells were harvested and the transcript pattern analyzed by RT-PCR. Depletion studies were also performed to verify the predicted changes for the SRSF1 (formerly ASF/SF2) splicing factor. Hep3B cells were firstly transfected with siRNAs targeting the mRNA of SRSF1 and luciferase (control) and 24 h later transfected with the WT or mutant c.257C>T minigenes. RT-PCR analysis was performed 48 h later. The sequences of all siRNAs are described in Table 1.

To confirm the overexpression of SRSF2, hnRNP E1 and hnRNP E2 as also the depletion of SRSF1 in whole cell lysates, quantitative Real-Time PCR (qRT-PCR) assays were performed. Relative levels of gene expression were analyzed using Taqman Universal PCR Master Mix 1x (Applied Biosystems) and the Taqman Gene Expression Kit (which includes primers and probes for each specific splicing factor gene – Table 1). The relative mRNA levels of target genes were calculated using standard curves (values ranging from 0.05 ng to 50 ng of RNA converted into cDNA). A standard curve was constructed for each target gene relating Ct values to log RNA quantities. The normalization of expression was given by the ratio between the RNA concentrations of each target gene and the endogenous gene *PGK1*. The relative amount of RNA was determined *via* the ratio of the normalized expressions of the target and control samples (Table 2).

The depletion of the SRSF1 splicing factor was also confirmed through Western blotting analysis. The immunodetection was carried out using the primary antibodies mouse anti-SF2/ASF clone 96 from Zymed (San Francisco, CA) and anti-α-tubulin from Sigma-Aldrich (Switzerland) (Fig. 4).

## Figures and Tables

**Table 1:** Description of the different sequences used in the work: primers for PCR amplifications^a^; small interfering RNA׳s for silencing of specific genes; probes used for real-time quantitative PCR; antisense oligonucleotide sequences.

**Oligonucleotide**	**Sequence (5′ to 3′)**
**Cloning fragments**	
Intron 1 BamHI F	ATATATGGATCCTCCAGCCTTGGGCCTCTT
Intron 3 XhoI R	ATATATCTCGAGGAATGCTGGATTCAGACA
Intron 7 IDS F	TATCTCGAGGAACCGCCACAGAGTCCT
Intron 8 IDS R	TATGGATCCGCACCTGTTCCTTTGTCC
**RT-PCR fragments**	
Exon 2 IDS F	TCATCATCGTGGATGACCTG
Exon 3 IDS R	AAAGACTTTTCCCACCGACA
Exon 7 IDS F	GGAAAATCCGCCAGAGCTAC
Exon 9 IDS R	GATCTCCACCTTGGGAATCA
**Plasmid vector primers**	
K-myc-Bam F	TACCGCCACCATGGAGCAGAA
pfGHr1 R	TTTATTAGGAAAGGACAGTGGG
SD6 F	TCTGAGTCACCTGGACAACC
SA2 R	ATCTCAGTGGTATTTGTGAGC
**Small interfering RNA׳s**	
siSRSF1(formerly ASF/SF2)-a	AGAAGAUAUGACCUAUGCA
siSRSF1 (formerly ASF/SF2)-b	GCAGGUGAUGUAUGUUAUG
siLUC (control)	CGUACGCGGAAUACUUCGA
**TaqMan gene expression assays**	
SRSF2 (formerly SC35)	Hs 00427515_g1 (Applied Biosystems)
SRSF1 (formerly ASF/SF2)	Hs 00199471_m1 (Applied Biosystems)
hnRNP E1 (PCBP1)	Hs 00362410_s1 (Applied Biosystems)
hnRNP E2 (PCBP2)	Hs 01590472_mH (Applied Biosystems)
**Antisense Oligonucleotides**	
AMO 1 IDS	GTAAGGGAAAAGCTTCTCACCTGCC
AMO 2 IDS	TTCTCACCTGCCTCCGGAAGTGAAG
AMO 3 IDS	AAAGCTTCTCACCTGCCTCCGGAAG
AMO Standard	CCTCTTACCTCAGTTACAATTTATA
LNA IDS	AAAGCTTCTCACCTGCCTCC


a Primers were designed according to the sequence described in the ENSEMBL database (www.ensembl.org; ENSG00000010404). F – Forward; R – Reverse.

**Table 2:** Quantification and normalization ratio for PGK1, SRSF2 (formerly SC35), hnRNP E1, hnRNP E2 and SRSF1 (formerly ASF/SF2) expression levels obtained through quantitative real-time PCR. Calculations of relative amounts of each target and endogenous reference RNA were determined using the appropriate standard curve.

**Sample**	**Amount of*****PGK1*****RNA (ng)**	**Amount of*****trans*****-acting factor RNA (ng)**	**Normalized amount of*****trans*****-acting factor RNA (ng)**	**Ratio**
**SRSF2 overexpression**				
WT ex3 minigene+Empty vector	1.2	1.4	1.2	1
c.257C>T mut+Empty vector	1.3	1.8	1.3	1
WT ex3 minigene+SRSF2	1.5	14.6	9.7	8.1
c.257C>T mut+SRSF2	1.2	17.9	14.6	11.2
**hnRNP E1 overexpression**				
WT ex3 minigene+Empty vector	0.45	0.2	0.45	1
c.257C>T mut+Empty vector	0.9	0.95	1.06	1
WT ex3 minigene+hnRNP E1	0.68	6.34	9.32	20.71
c.257C>T mut+hnRNP E1	0.46	8.73	18.98	17.91
**hnRNP E2 overexpression**				
WT ex3 minigene+Empty vector	0.76	0.22	0.29	1
c.257C>T mut+Empty vector	1.03	0.78	0.76	1
WT ex3 minigene+hnRNP E2	0.96	2.04	2.13	7.34
c.257C>T mut+hnRNP E2	0.76	3.89	5.12	6.74
**SRSF1 silencing**				
siLUC+WT ex3 minigene	0.58	0.55	0.95	1
siLUC+c.257C>T mut	0.99	0.77	0.78	1
siSRSF1+WT ex3 minigene	1.16	0.15	0.13	0.14
siSRSF1+c.257C>T mut	0.56	0.08	0.14	0.18
